# Filter-Based Dispersion-Managed Versatile Ultrafast Fibre Laser

**DOI:** 10.1038/srep25995

**Published:** 2016-05-17

**Authors:** Junsong Peng, Sonia Boscolo

**Affiliations:** 1 Aston Institute of Photonic Technologies, School of Engineering and Applied Science, Aston University, Birmingham B4 7ET, United Kingdom

## Abstract

We present the operation of an ultrafast passively mode-locked fibre laser, in which flexible control of the pulse formation mechanism is readily realised by an in-cavity programmable filter the dispersion and bandwidth of which can be software configured. We show that conventional soliton, dispersion-managed (DM) soliton (stretched-pulse) and dissipative soliton mode-locking regimes can be reliably targeted by changing the filter’s dispersion and bandwidth only, while no changes are made to the physical layout of the laser cavity. Numerical simulations are presented which confirm the different nonlinear pulse evolutions inside the laser cavity. The proposed technique holds great potential for achieving a high degree of control over the dynamics and output of ultrafast fibre lasers, in contrast to the traditional method to control the pulse formation mechanism in a DM fibre laser, which involves manual optimisation of the relative length of fibres with opposite-sign dispersion in the cavity. Our versatile ultrafast fibre laser will be attractive for applications requiring different pulse profiles such as in optical signal processing and optical communications.

Passively mode-locked fibre lasers have been intensively investigated due to their potential for realising reliable and cost-effective compact ultrafast light sources^1^,^2^,^3^. Nonlinear effects are usually quite large in mode-locked fibre lasers, but the interplay among the effects of gain/loss, dispersion and nonlinearity can also be used to shape the pulses and manipulate and control the light dynamics and, hence, lead to different regimes of mode locking. When the group-velocity dispersion (GVD) of the laser is anomalous, pulses form by a balance between positive nonlinear and negative dispersive phase changes, and the resulting pulse propagates indefinitely without change. Before 1993, researchers operated fibre lasers almost exclusively with large net anomalous GVD, in the soliton-like regime^4^,^5^. The energy achievable in such laser systems is limited to some tens of picojoules in single-mode fibre (SMF) by the soliton area theorem. In a laser with segments of large and nearly equal magnitudes of GVD but with opposite signs (referred to as a dispersion map), a pulse will stretch and compress and the nonlinear phase is exactly balanced by the net effect of dispersion. This stretched-pulse or dispersion-managed (DM) soliton operation^6^,^7^,^8^ exists for net anomalous or small normal GVD and allows ultrashort pulses with up to nanojoule energies. Recent work^9^ has shown that much higher pulse energies can be achieved in fibre lasers that operate at large normal dispersion. In the normal-dispersion regime, to compensate nonlinear phase and avoid wave-breaking phenomena, dissipation is required and plays a key role in the pulse shaping. The similariton regime existing in DM cavities with large normal GVD features parabolic pulses that evolve self-similarly in a long segment of passive fibre^10^. These pulses linearise the nonlinear phase in the normal-dispersion fibre, which is compensated by an anomalous-GVD segment. Parabolic amplifier similaritons have also been stabilised in a fibre oscillator^11^,^12^,^13^,^14^. The amplifier similariton laser relies on a local nonlinear attraction to stabilise the pulse in the cavity, while spectral filtering plays an important supporting role by undoing the large spectral broadening after the gain segment. It is worth to mention that the soliton-similariton laser reported in refs 11 and 12 is the only example of mode-locked laser so far that has two types of nonlinear waves propagating within the cavity. In cavities with only normal-dispersion components or strong net normal dispersion^15^,^16^,^17^,^18^,^19^, the resulting dissipative solitons (DSs) balance nonlinear phase accumulations by spectral or gain filtering of a highly chirped pulse in the laser cavity. To date, the best energy performance from SMF lasers has been achieved with this mode-locking mechanism.

Techniques for generating, controlling and manipulating ultrashort optical pulses and specialised waveforms^20^ have become increasingly important in many scientific areas, including, amongst others, ultrahigh-speed optical communications, optical signal processing and bio-photonics. Versatile ultrafast laser sources, which can selectively emit different types of nonlinear waves, are highly desirable in this context. Different regimes of pulse generation at net anomalous or normal dispersion can be realised in a DM fibre laser via appropriate in-cavity dispersion management^21^,^22^,^23^,^24^. Commonly employed methods to achieve flexible control of in-cavity dispersion include grating pairs^24^,^25^, prisms, or simply physically changing the length of the fibre in the cavity^21^,^22^,^23^. Furthermore, the possibility to achieve both parabolic self-similar and triangular pulse shaping in a mode-locked fibre laser via adjustment of the net normal dispersion and integrated gain of the cavity was reported in ref. 26. In ref. 27, careful control of the gain/loss parameters of a net-normal dispersion laser cavity provided the means of achieving switching among Gaussian pulse, DS and similariton pulse solutions in the cavity. All these techniques however, require manual tuning of some physical parameters of the cavity.

Spectral pulse shaping^28^ is a technique that employs spectral manipulation of the intensity and phase components of a pulse in order to create the desired field distribution. Recently, the inclusion of a spectral pulse shaper into the cavity of a mode-locked fibre laser has emerged as a method to achieve a potentially high degree of control over the dynamics and the output of the laser^29^,^30^,^31^,^32^,^33^,^34^, while obviously entailing a more power efficient technique than pulse shaping implemented through direct filtering of a laser output. Fully programmable phase and amplitude filters^35^ are already commercially available and extensively used in telecommunications applications^36^. The use of such filters, when placed inside a laser cavity, has the potential to allow the operation of lasers that exhibit pulse characteristics that can be controlled purely through software control. This method was demonstrated to allow for tuneability of the laser wavelength and laser operation at high repetition rates^29^. In refs 30 and 31, the phase-filtering ability of an in-cavity pulse shaper was shown to enable precise control of the cavity dispersion of the laser as well as to change the output pulse train from bright to dark pulses. In ref. 32, it was numerically shown that a passively mode-locked fibre laser can operate in different pulse-shaping regimes, including bright and dark parabolic, flat-top, triangular, and saw-tooth waveform generations, depending on the amplitude profile of an in-cavity spectral filter. Furthermore, an application of this technique using a flat-top spectral filter was numerically demonstrated to achieve the direct generation of high-quality sinc-shaped optical Nyquist pulses with widely tuneable bandwidth from a passively mode-locked fibre laser^33^. An experimental demonstration of Nyquist pulse generation from a regeneratively and harmonically mode-locked fibre laser with a spectral pulse-shaping filter installed in the cavity was reported in ref. 34.

In this paper, we report on a versatile erbium-doped fibre (EDF) laser in which soliton, DM soliton, and DS mode-locking regimes can be selectively and reliably established by programming different GVD profiles and bandwidths on an in-cavity programmable filter. The generation and in-cavity evolution of the different pulses are confirmed by a numerical analysis. We would like to note that the aim of our proof-of-concept experiment is not to achieve laser output performances that exceed those of standard fibre lasers. Instead, we aim to demonstrate the novel possibility of adaptively controlling the pulse formation mechanism in a mode-locked fibre laser by inclusion of a programmable filter in the laser cavity. Each of the mode-locking regimes achieved in our laser obviously takes on major practical importance. For example, soliton lasers are finding widespread applications as absolutely turnkey ultrafast laser sources in industrial environments. DM solitons are very attractive for super-continuum generation owing to their ultrashort pulse duration and broad spectrum^37^. DSs with high energy and chirp can simplify chirped-pulse amplification systems when serving as a seed source^16^. To our knowledge, this is the first time that these distinctly different pulse solutions are obtained in a single laser system without applying any physical changes in the layout of the laser cavity. The programmable-filter-based technique presented in this paper greatly relaxes the need for employing opposite-sign dispersion fibres and fibre length optimisation in order to achieve the desired pulse-shaping regime in ultrafast fibre lasers.

## Results and Discussion

### Setup and experimental results

**Figure 1:**
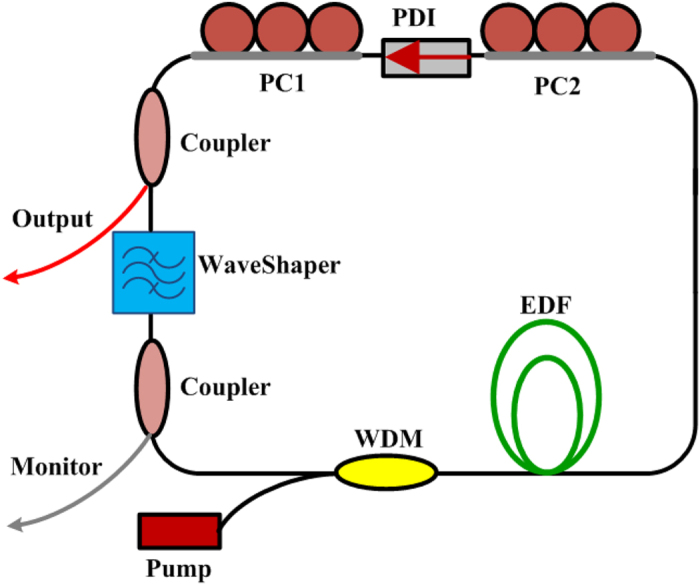
Schematic of filter-based dispersion-managed passively mode-locked EDF laser. WDM: wavelength-division multiplexer; EDF: erbium-doped fibre; PC: polarisation controller; PDI: polarisation-dependent isolator.

**Figure 2:**
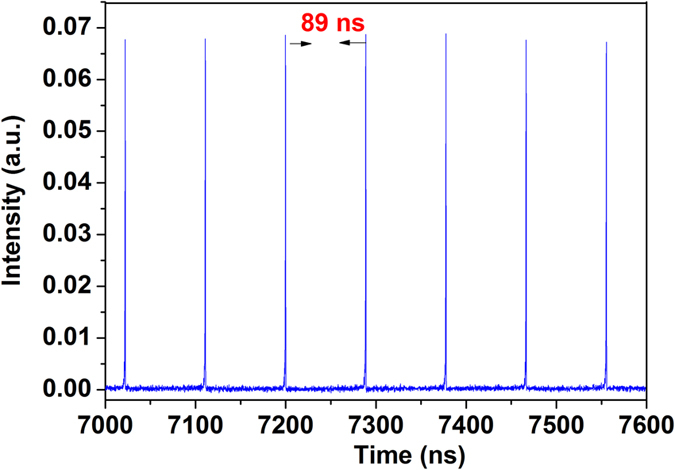
Typical output pulse train of the mode-locked fibre laser showing a pulse spacing of 89 ns.

The laser setup in the ring cavity configuration is shown in Fig. 1. A 1.2-m long segment of EDF with nominal absorption coefficient of ~52 dB/m at 976 nm and normal GVD *β*_2_ = 65.05 fs^2^/mm at 1550 nm was used as the gain medium. This fibre was pumped through a 980/1550 wavelength-division multiplexer (WDM) by a 976-nm laser diode, which could provide up to 300 mW optical power. An in-fibre polarisation-dependent isolator (PDI) sandwiched with two polarisation controllers (PCs), converted nonlinear polarisation rotation (NPR) to amplitude modulation, initiating and stabilising mode-locked operation^38^,^39^, and it also ensured single direction oscillation. Additional anomalous-dispersion fibre associated with the various components of the laser cavity amounted to a length of ~8 m. A programmable liquid-crystal-on-silicon (LCoS) phase and amplitude filter^35^ was used to realise different regimes of pulse generation in the cavity. (Further details can be found in the Methods.) Under mode-locking conditions, the laser operated at a repetition period of 89 ns (Fig. 2), which remained constant throughout the experiments described below (since there was no change in the optical length of the cavity). The net dispersion of the cavity was adaptively managed by changing the curvature of the parabolic spectral phase profile of the filter (see Methods). As pulse formation at net anomalous or slightly normal GVD does not depend on spectral filtering, for such dispersion regimes the amplitude filtering ability of the filter was not used: we employed the default flat-top filter’s shape with the maximum achievable bandwidth of 9 THz (73 nm). On the other hand, for operation of the laser in the strong net-normal dispersion regime, the filter was programmed to have a narrow-bandwidth Gaussian spectral profile, as detailed below. A filter’s central wavelength of 1555 nm was used for all dispersion regimes at play. Two 90:10 fibre couplers were employed to tap 10% of laser power out of the cavity, where one was for laser outputs after the NPR components; the other one was located after the programmable filter for investigating the pulse dynamics inside the laser cavity. The output port was connected by a 3-m long segment of anomalous-dispersion SMF (*β*_2_ = −22.8 fs^2^/mm) to an optical spectrum analyser, an auto-correlator and a fast photodetector (1-GHz bandwidth) connected to an oscilloscope (2-GHz bandwidth) to characterise the pulses.

**Figure 3:**
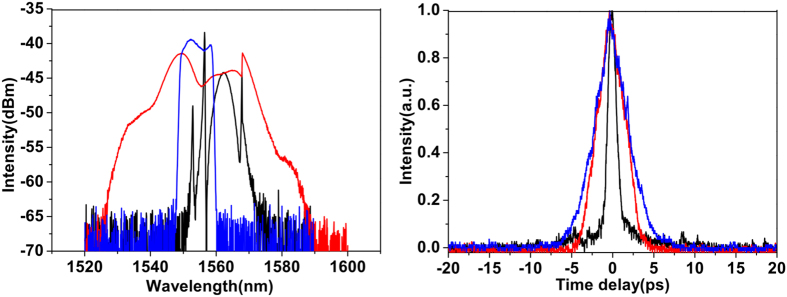
Typical measured optical spectra (left) and autocorrelation traces (right) for the output soliton (black), DM soliton (red) and DS (blue).

**Figure 4:**
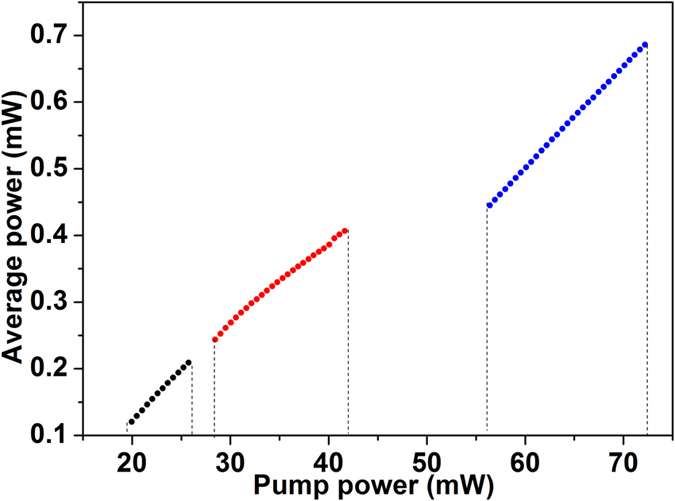
Average output power from the laser as a function of pump power for single-pulse soliton (black), DM soliton (red) and DS (blue) mode locking.

When there was no dispersion applied on the programmable filter, the cavity had an anomalous net dispersion. By properly adjusting the two PCs in the system, stable mode-locked pulses could be obtained. A typical pulse train as observed on the oscilloscope is shown in Fig. 2. In this example, the average pulse power after the SMF of the laser output port was 0.15 mW. The black curve in the left panel of Fig. 3 shows the corresponding optical spectrum profile at the laser output, centred at ~1560 nm and with a spectral bandwidth at full-width at half-maximum (FWHM) of 4.7 nm. The presence of distinct Kelly sidebands^40^ in the spectrum indicates the fundamental soliton shape of the output pulse. The net dispersion of the cavity, calculated from the sidebands, is −0.3967 ps^2^ (at 1555 nm). Note that because the filter bandwidth is wide and the filter’s shape is flat, the filter has negligible effect on soliton generation; thereby the central wavelength of the soliton is only determined by gain/loss in the laser and deviates from that of the filter. The black curve in the right panel of Fig. 3 shows the measured autocorrelation trace of the pulse corresponding to a FWHM pulse duration of 0.8 ps when a hyperbolic secant fit is assumed. This gives a time-bandwidth product (TBP) of 0.46 (at 1560 nm), indicating that the pulse is slightly chirped. We attribute it to the GVD of the SMF of the laser output port. The soliton-like single-pulse generation regime was observed for a pump power of 20 to 26 mW (Fig. 4), and bore an average output power of 0.12 to 0.21 mW corresponding to a pulse energy 10.7 to 18.7 pJ. When some normal dispersion was applied on the filter while keeping the net cavity dispersion anomalous, the spectral width of the pulses increased, which is in agreement with soliton-type pulse behaviour^41^.

Next, we applied a dispersion of 0.385 ps^2^ (at 1555 nm) on the filter to shift the net cavity GVD close to zero value. In this case, after adjustment of the PCs, the laser generated pulses (Fig. 3, red curves) with Kelly sideband-free, wide spectrum, which is a signature of the DM soliton operation regime. The spectral width at FWHM is around 25 nm. An even wider output pulse spectrum was obtained by establishing the net GVD to be slightly normal^41^ (0.014 ps^2^). Single-pulse DM soliton mode locking existed under a pump power of 28.5 to 42 mW (Fig. 4), and bore an average output power of 0.24 to 0.41 mW, yielding a pulse energy of 21 to 36 pJ, which is significantly higher than that of soliton-like mode locking. The autocorrelation trace of the pulse with 0.20 mW average power after the SMF of the laser output port indicates a FWHM pulse duration of 2.78 ps when a Gaussian fit is assumed, which results in a TBP of 8.62 (at 1555 nm).

It is well known that DSs occur in all-normal^15^,^16^ or strong net-normal^17^,^18^,^19^ dispersion cavities. In our laser design, strong net-normal dispersion can be achieved by configuring the dispersion of the in-cavity filter; meanwhile, the filter bandwidth also needs to be properly controlled as DSs require additional amplitude modulation besides saturable absorber action^9^,^15^. Furthermore, a Gaussian spectral profile was programmed on the filter, as the default flat-top profile is similar to the shape of the DS spectrum. Self-starting mode-locked operation was achieved by adjustment of the PCs with a filter bandwidth at FWHM of 0.8 THz. No stable mode-locked pulses were found for larger bandwidths, consistently with the discussion above. The blue curves in Fig. 3 give the characterisations of the laser output obtained for a filter’s dispersion of 0.8986 ps^2^, yielding a net GVD of 0.502 ps^2^ in the cavity, and 0.48-mW average pulse power after the SMF of the output port. The output pulse spectrum is steep at the edges and has a dip in the top, typical characteristics of DS lasing^9^,^15^. The spectral width at FWHM is 9.7 nm. Incidentally, we also note that the spectrum is centred at the central wavelength of the filter, which confirms the ability of an in-cavity programmable filter to tune the laser wavelength^29^. The pulse autocorrelation trace corresponds to a FWHM pulse duration of 3.5 ps when a Gaussian fit is assumed. This gives a TBP of 4.21 (at 1555 nm), indicating that the pulse is highly chirped inside the cavity^9^,^15^. The average output power for the DS mode-locking regime was 0.45 to 0.69 mW under the pump power 56.5 to 72 mW (Fig. 4), giving a pulse energy 39 to 61 pJ. Laser generated DSs were found to exist in the net cavity dispersion range 0.245 to 0.502 ps^2^, and their spectral width decreased when the in-cavity GVD was increased, as it was expected.

In Fig. 4 we summarised results for the scaling of the laser output power with pump power for the different pulse-generation regimes. It is seen that the DS regime features the highest pumping threshold for mode locking and, correspondingly, the highest output pulse energy, in accordance with previous findings. The efficiency of power transfer from the pump to the laser signal, indicated by the slope of the curves in Fig. 4, is also highest for the DS regime, owing to the large chirp of the pulses in the cavity. At a first glance it might seem strange that the soliton regime exhibits higher power transfer efficiency than DM soliton lasing. However, this accords well with the fact that the DM soliton spectrum is so wide that it saturates the gain bandwidth of the EDF; this in turn results in less efficiency. For each pulse-generation regime and a fixed dispersion applied on the programmable filter, increasing the pump power beyond the maximum value bearable by single-pulse mode locking resulted in multiple pulse generation, which also led to pulse bound states. These regimes of emission have been intensively investigated in fibre lasers with anomalous and normal net cavity dispersion^42^,^43^,^44^. When the pump power was increased further the laser operated in a noise-like pulsing mode. It is also to be appreciated that when the laser operates in a given pulse-shaping regime, changing the net cavity GVD does not destroy mode locking as long as the laser is still in its original regime. However, switching among the different regimes needs adjustment of the PC settings and pump power. On the other hand, the laser can no longer be mode locked when the cavity dispersion is larger than the upper boundary of the dispersion region where DSs exist in the system.

### Numerical simulations

To gain insight into the pulse dynamics inside this fibre laser cavity, we performed numerical simulations of the laser based on a non-distributed model solving every part by the nonlinear Schrödinger equation. The laser configuration used in the simulations was the same as the experimental setup. To account for additional length of anomalous-dispersion fibre in the cavity, we included two segments of SMF after the saturable absorber (SA) element and the filter, respectively. Parameters were chosen to match the experimental values. Further details can be found in the Methods.

**Figure 5:**
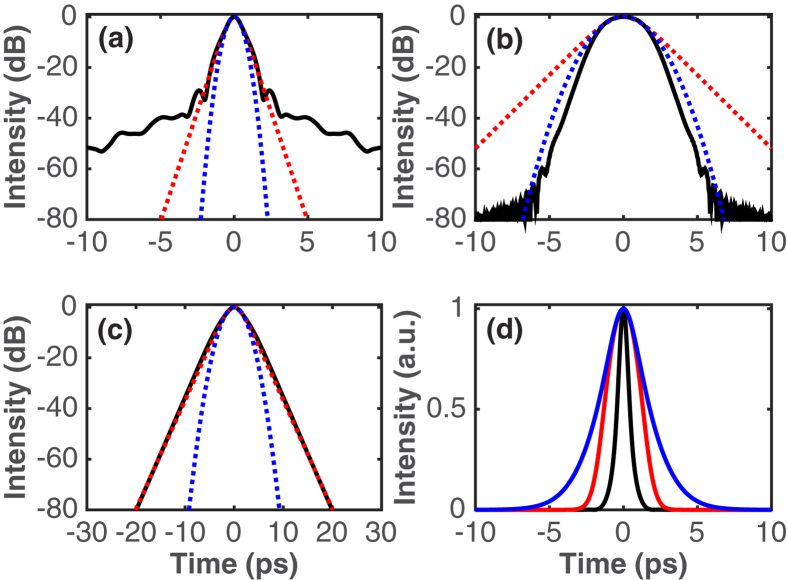
Simulated temporal intensity profile of the pulse at the output of the laser operating in the (**a**) soliton, (**b**) DM soliton, and (**c**) DS generation regimes on a logarithmic scale. Also shown are hyperbolic secant (dotted red) and Gaussian (dotted blue) fits to the pulse shape. (**d**) Temporal intensity profiles for the soliton (black), DM soliton (red), and DS (blue) regimes on a linear scale.

**Figure 6:**
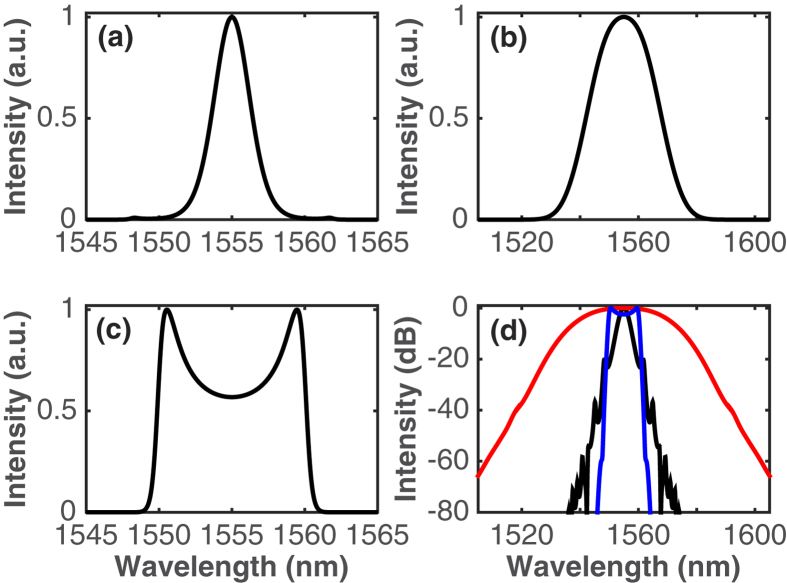
Simulated spectral intensity profile of the pulse at the output of the laser operating in the (**a**) soliton, (**b**) DM soliton, and (**c**) DS generation regimes on a linear scale. (**d**) Spectral intensity profiles for the soliton (black), DM soliton (red), and DS (blue) regimes on a logarithmic scale.

**Figure 7:**
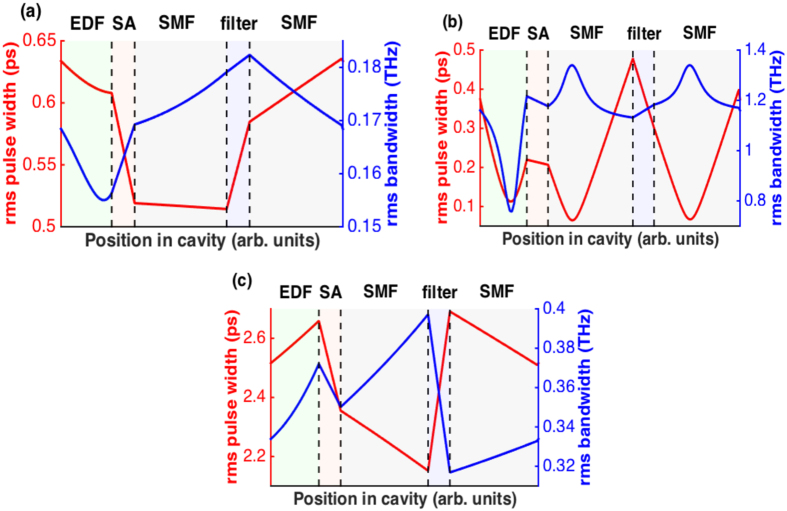
Simulated evolution of the rms temporal (red) and spectral (blue) widths of the pulse along the cavity for the (**a**) soliton, (**b**) DM soliton, and (**c**) DS regimes.

Example pulse solutions obtained after the SMF of the laser output port in the three pulse-generation regimes are shown in Figs 5 and 6, showing the simulated pulses and spectra, respectively. The respective net GVD of the cavity was −0.397 ps^2^, 0 ps^2^ and 0.502 ps^2^, which are similar values to those of the experimental results in Fig. 3. There is a qualitative fairly good agreement between the simulated and experimental pulse modes of operation and spectral profiles. Furthermore, the temporal durations of the simulated pulses agree well with those measured in the experiment. The soliton-like pulse has FWHM temporal and spectral widths of 0.88 ps and 2.87 nm, yielding a TBP of 0.314, which almost equals the transform limit for a hyperbolic secant pulse; the temporal and spectral widths of the DM soliton pulse are 2.64 ps and 26.1 nm, yielding a TBP of 8.53, and the DS features 3.57-ps pulse width and 10.3-nm bandwidth, thus a TBP of 4.54. These values are close to the experimental ones. The in-cavity pulse evolution is illustrated by plots of the root-mean-square (rms) pulse duration and spectral bandwidth as functions of position in the cavity (Fig. 7). Distinctly different types of evolutions can be seen from this figure. The soliton-like pulse is nearly static in the cavity, with both temporal and spectral breathing ratios (defined as the ratios of maximum and minimum rms widths within a round-trip in the cavity) of only 1.2. The DM soliton experiences the largest temporal and spectral breathing, with the respective breathing ratios 7.5 and 1.8. This is typical of the DM mode of operation^45^. This pulse temporally stretches and compresses three times per round-trip, reaches a minimum duration in the middle of each of EDF and two SMF segments, and acquires both signs of chirp. The fact that in our laser cavity the pulse stretches and compresses three times per round-trip instead of twice as within typical DM soliton fibre lasers^6^,^7^,^8^ stems from the presence of a spectral filter with normal dispersion between two segments of fibre with anomalous dispersion. Indeed, the filter imparts a positive chirp onto the negatively chirped pulse at the exit of the first anomalous segment, thereby temporally compressing the pulse. The resultant positively chirped pulse experiences further compression in the second anomalous segment up to the point where the chirp is completely cancelled. The mode-locked pulse at large net-normal dispersion exhibits the features of a DS with an evolution defined by the dispersion map^18^. The evolution in each fibre segment is monotonic, the pulse duration increases in the EDF segment and decreases in the SMF segments, and the spectrum, cut away by the filter, grows back in the fibre sections. The pulse is positively chirped throughout the cavity, and it depends strongly on dissipative effects such as the spectral filter and the SA. The different roles played by spectral filtering in the DM soliton and DS mode-locking regimes are evident: in the DM soliton regime, the large filter has nearly no effect on the pulse spectrum, while the pulse is temporally compressed by the effect of the normal dispersion applied on the filter. In the DS regime, by contrast, the narrow filter cuts away the spectral structure and, consequently, the pulse duration is increased.

## Conclusion

At the level of fundamental research, mode-locked lasers constitute an ideal platform for the investigation of original and complex nonlinear dynamics of ultrashort pulses, while at the applied research level, pulses with different and optimised features – e.g., in terms of pulse duration, temporal and/or spectral shape, width, energy, repetition rate and emission bandwidth – are sought with the general constraint of developing efficient cavity architectures. In these two aspects, fibre laser cavities, which offer compact and versatile design, represent an area of intense development. A number of experimental demonstrations have proved the ability of an in-cavity spectral pulse shaper based on a programmable optical processor to provide a high degree of control over the dynamics and the output of mode-locked fibre lasers^29^,^30^,^31^,^34^. A completely different method to externally control the in-cavity dynamics of mode-locked fibre lasers, based on using an evolutionary algorithm to search pulsed regimes in the laser cavity, has also been recently reported^46^. Our work fits into this research area through the development of a versatile ultrafast passively mode-locked EDF laser in which the pulse formation mechanism can be flexibly controlled by an in-cavity programmable filter. We have demonstrated for the first time to the best of our knowledge that conventional solitons, DM solitons and DSs can be selected and reliably targeted in a single laser by adaptively changing the dispersion and bandwidth programmed on the in-cavity filter. We have confirmed the different regimes of pulse generation experimentally and by numerical simulations. The transitions among the different pulse-shaping regimes are easily reconfigurable, and inherently interesting in the field of ultrafast optics owing to their vastly different characteristics. The combination of two mature technologies – linear shaping and fibre laser – demonstrated in this paper offers new technological opportunities and paves the way for the generation of more advanced waveforms. This multifunction ultrashort-pulse fibre laser source has a great potential for several applications, such as optical communications and signal processing, and can also be readily implemented at different wavelength regions, such as at the two microns region, where suitable dispersion-compensating fibres are still under development. We also emphasize that linear shaping is now readily available at one micron for the ytterbium-fibre-based technology. This could be exploited to scale up the output energy of the laser. Moreover, as far as the energy performance enhancement is concerned, our laser could be used as the master laser in a master-oscillator-power-amplifier (MOPA) system. In a MOPA configuration, the possibility of changing the operating regime of the seed laser may be interesting for engineering the following high-gain amplification stage.

## Methods

Numerical simulations of pulse propagation within the fibre sections were based on a standard modified nonlinear Schrödinger equation^47^:


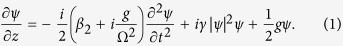


Here *ψ* = *ψ*(*z, t*) is the slowly-varying amplitude of the pulse envelope, *z* is the propagation coordinate, *t* is the time delay parameter, *β*_2_ is the GVD parameter and *γ* is the coefficient of cubic nonlinearity for the fibre section. The dissipative terms represent linear gain as well as a parabolic approximation to the gain profile with the bandwidth Ω. The gain is described by *g* = *g*(*z*) = *g*_0_/(1 + *W*/*W*_0_), where *g*_0_ is the small-signal gain (corresponding to 30 dB/m in power), which is non-zero only for the gain fibre, 
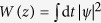
 is the pulse energy, and *W*_0_ is the gain saturation energy determined by the pump power. To initiate and sustain mode locking of the fibre laser, the NPR technique was used in our experiment. Here the mode-locking regime for the sake of clarity was modelled by a simple transfer function^47^: *T* = 1 − *q*_0_ − *q*_*m*_/[1 + *P*(*t*)/*P*_0_], where *q*_0_ is the unsaturated loss due to the absorber, *q*_*m*_ is the saturable loss (modulation depth), *P*(*z, t*) = |*ψ*(*z, t*)|^2^ is the instantaneous pulse power, and *P*_0_ is the saturation power. The filter was modelled by the spectral response^32^: *H*(*f*) = *R*(*f*)exp[*iβ*_2,acc_(2*πf*)^2^/2], where the spectral phase added a specific amount of GVD *β*_2,acc_ (in ps^2^) to the cavity to control the net cavity dispersion. We used a rectangular spectral profile *R*(*f*) = rect(*f*/*B*) with the bandwidth *B* = 9 THz to operate the laser at anomalous or slightly normal dispersion, and a Gaussian spectral profile *R*(*f*) = exp[−*f*^2^/(2*B*^2^)] with a FWHM bandwidth of 0.8 THz for the strong net-normal dispersion regime, in accordance with the experiment. Linear losses of 10% were imposed after the SMF segment following the SA and after the filter, which summarised intrinsic losses and output coupling. The laser output was monitored behind the coupler at the exit of the SMF segment following the SA, after further propagation in a 3-m-long segment of SMF as in the case of the experiment. The parameters used in the numerical simulations were similar to their nominal or estimated experimental values. We would like to point out that we did not aim here at a comprehensive comparison of numerical modelling and experiments and intentionally considered a simplified description of some key effects. Instead, we used this simple model to highlight the main features of the generated pulse propagation regimes. The numerical model was solved with a standard symmetric split-step propagation algorithm, and the initial field was a picosecond Gaussian temporal profile.

Experimentally, the spectral filter was provided by the commercially available wave-shaper Fourier-domain programmable optical processor, which allowed for easy reconfiguration of the spectral filter. A detailed description of the Fourier-domain programmable optical processor is given in ref. 35; suffice to say here that it works by diffracting the input light onto a two-dimensional LCoS array. Applying phase variations across either the horizontal or vertical dimension of the array controls the spectral phase and the intensity, respectively. The bandwidth of the device is programmable in 1 GHz increments from 20 GHz up to the whole C + L band.

## Additional Information

**How to cite this article**: Peng, J. and Boscolo, S. Filter-Based Dispersion-Managed Versatile Ultrafast Fibre Laser. *Sci. Rep.* **6**, 25995; doi: 10.1038/srep25995 (2016).
